# Framing health taxes: learning from low- and middle-income countries

**DOI:** 10.1136/bmjgh-2023-012955

**Published:** 2023-10-13

**Authors:** Kaung Suu Lwin, Adam D Koon, Kumanan Rasanathan, Abdillah Ahsan, Daniel Erku, Melissa Mialon, Silvana Perez-Leon, Arti Singh, Zafar Mirza, Mario Zuleta, Shiva Raj Adhikari, Yubraj Acharya, Son The Dao, Sabrina Rasheed, Jeremias Paul, Robert Marten

**Affiliations:** 1 Alliance for Health Policy and Systems Research, Geneva, Switzerland; 2 Department of International Health, Johns Hopkins Bloomberg School of Public Health, Baltimore, Maryland, USA; 3 Department of Economics, Facutly of Economics and Business, University of Indonesia, Depok, Indonesia; 4 Institute of Public Health, University of Gondar, Gondar, Ethiopia; 5 Centre for Applied Health Economics, Griffith University, Gold Coast, Queensland, Australia; 6 Trinity College Dublin, Dublin, Ireland; 7 CRONICAS Center of Excellence in Chronic Disease, Universidad Peruana Cayetano Heredia, Lima, Peru; 8 School of Public Health, KNUST, Kumasi, Ghana; 9 School of Universal Health Coverage, Shifa Tameer-i-Millat University, Islamabad, Pakistan; 10 City University of London, London, UK; 11 Central Department of Economics, Tribhuvan University, Kathmandu, Nepal; 12 Department of Health Policy & Administration, The Pennsylvania State University, University Park, Pennsylvania, USA; 13 Thuongmai University, Ha Noi, Vietnam; 14 Health Systems and Population Sciences Division, International Centre for Diarrhoeal Disease Research, Dhaka, Bangladesh; 15 Fiscal Policies for Health Unit, Department of Health Promotion, WHO Secretariat, Geneva, Switzerland

**Keywords:** Health policy, Health systems

## Abstract

Health taxes are effective policy instruments to save lives, raise government revenues and improve equity. Health taxes, however, directly conflict with commercial actors’ interests. Both pro-tax health advocates and anti-tax industry representatives seek to frame health tax policy. Yet, little is known about which frames resonate in which settings and how framing can most effectively advance or limit policies. To fill this gap, we conducted qualitative research in 2022, including focus group discussions, in-depth interviews, document reviews and media analysis on the political economy of health taxes across eight low-income and middle-income countries. Studies captured multiple actors constructing context-specific frames, often tied to broader economic, health and administrative considerations. Findings suggest that no single frame dominates; in fact, a plurality of different frames exist and shape discourse and policymaking. There was no clear trade-off between health and economic framing of health tax policy proposals, nor a straightforward way to handle concerns around earmarking. Understanding how to best position health taxes can empower health policymakers with more persuasive framings for health taxes and can support them to develop broader coalitions to advance health taxes. These insights can improve efforts to advance health taxes by better appreciating political economy factors and constraining corporate power, ultimately leading to improved population-level health.

**Summary box**

- There is growing evidence demonstrating health taxes’ impact as an effective measure not only to improve health and well-being of the population but also to raise government revenues.
- However, there is limited evidence on framing and the political economy of health taxes, especially in low-income and middle-income countries (LMICs).
- The most common arguments in support of health taxes are that they can reduce suffering and premature death and generate additional revenue streams for governments. Arguments that did not surface as prominently were arguments based on equity, benefits to the economy or international precedent. Arguments against health taxes largely focused on their potential to hurt the economy and promote illicit trade.
- This research from LMICs suggests no single frame dominated policy processes for health taxes; instead, multiple frames interact and shape health tax policy.
- Health tax policy in LMICs continues to be informed largely by experiences passing and adjusting tobacco taxes, while newer health taxes such as sugar-sweetened beverages remain understudied.
- Researchers, advocates and policymakers may generate greater support for health taxes by developing multiple frames that resonate with different types of values rather than searching for a single strong frame that is universally applicable across contexts.
- Insights into this study provide a richer understanding of the political forces that shape health tax narratives in service of better policy design and implementation.

## Introduction

Around 41 million people died globally of preventable non-communicable diseases (NCDs) in 2019.^1^ Consumption of harmful products, such as tobacco, alcohol and sugar-sweetened beverages (SSBs), contributes to NCDs.^2^ In fact, just four industry sectors (ie, tobacco, ultra-processed food, fossil fuel and alcohol) account for at least a third of global deaths.^3^ Taxes reducing the consumption of these products improve health, save lives and generate additional revenue for health and developmental agendas.^4^ Yet health taxes remain vastly underutilised, both in terms of implementation rates and in terms of the products that are taxed.^5^

Applying policy and political economy analysis (PEA) can help policymakers understand why progress deploying this proven intervention is limited. PEA focuses attention on power dynamics by tracing both visible short-term efforts to compel action and invisible long-term efforts to define interests.^6^ In this way, PEA can help improve implementation and can also contribute to advancing health taxes by elucidating the social construction of arguments or frames to overcome policy barriers. Frames are collective ways of narrating and understanding the world.^7^ PEA case studies from Mexico, Chile and Colombia and other countries show how different health and economic frames shape health tax policy.^8–13^ What is less clear, however, is the interplay of frames for and against health taxes and which arguments resonate in which settings or with which types of stakeholders.^13 14^ Broader socioeconomic forces can shape political climates and make some arguments more attractive. Identifying the best framing, and frame sponsor, could help catalyse coalitions and compel policymakers to take political action to advance health taxes.^14^ It may also reveal the complex ways in which power is exercised and institutionalised through public discourse. For example, as Babor, Collin and Monteiro argue ‘framing health taxes in terms of their economic, social, and public health benefits rather than allowing industry to define them as a liability can be a persuasive argument that could increase the chances of implementing effective NCD prevention’.^15^ To improve policy design and accelerate health tax implementation, governments need to understand the best way to position and frame health taxes as part of the overall process of policy development and implementation.^15–17^

Libertarians believe taxes infringe on individual freedom and are a funding source for wasteful government spending.^17 18^ Individualists may support health taxes to pay for associated social costs.^17 18^ Health taxes are highly visible policy mechanisms, and politically contested.^12 17 18^ Taxes, and the revenue they generate, can also be understood as mechanisms to support and fund social programmes which are consistently popular with voters.^12 17 18^ They can also be perceived to limit macroeconomic growth and constrain domestic labour markets, making them unpopular with key domestic industries.^18–21^ Within government, different ministries/departments, committees and individual representatives hold conflicting views on health taxes, which are often coloured by their relationships with different industries. For this reason, health taxes sometimes feature in elections as part of political party manifestos and campaign platforms.^14^ Moreover, NCD control measures, such as taxation, surface at overlapping political jurisdiction - from national elections to city council races.^12–15^

Government, private sector and civil society actors shape NCD prevention and control.^12–18^ Political funding and messaging from industry-related stakeholders, such as potential price increases and job losses, may obstruct NCD prevention and control efforts such as health taxes.^19–21^ In this way, a key source of corporate power is the ability to frame commercial activity in ways that erode regulatory authority and lead to the proliferation of harmful products.^22^ Health taxes need to be understood within the context of the broader impacts of the private sector on health (the ‘commercial determinants of health’) to fully appreciate the economic, social and political factors which contribute to or limit their implementation.^3^ Addressing complex commercial determinants requires multisectoral collaboration and coproduction of different kinds of knowledge to build support for social mobilisation and policy change.^3 16 23 24^

To support these efforts, the Alliance for Health Policy and Systems Research, with support from the Government of Norway, launched a research programme in 2021 to explore the political economy of health taxes in low-income and middle-income countries (LMICs).^5^ Case study research was conducted in eight countries: Bangladesh, Ethiopia, Ghana, Indonesia, Nepal, Pakistan, Peru and Vietnam (table 1). All case studies were led by researchers from the respective LMIC. Consistent with interpretive research methods from policy studies, each case was nested in specific country and policy contexts. Each selected a different tax or set of taxes, historical reference points and used slightly different qualitative study designs. Most made heavy use of media content analysis, others conducted key informant interviews. All used some form of document review. The diversity in case study design enabled attention to local context and enriched analysis. All studies focused on the political dimensions of health tax policy change, with a particular focus on the political interplay between actors and their arguments to support or oppose tax policies. This study starts by summarising lessons from individual case studies, continues with learning across case studies; it continues with potential lessons for global efforts to advance health taxes and concludes with an agenda for action and an agenda for research.

**Table 1:** Characteristics of country studies

Country	Authors	Health taxes under analysis	Data source(s) used for analysis	Arguments for health taxes	Arguments against health taxes	Outcome of policy process (at time of research)
Bangladesh	Rasheed *et al*	Alcohol, tobacco	News media, government documents, key informant interviews	Reduces suffering and premature death, lucrative for governments	Hurts/eliminates jobs, threat to industry, promote illicit trade	Contestation (unresolved)
Ethiopia	Erku *et al* 27	Tobacco	News media, government documents, legislative proceedings, focus group discussions, key informant interviews	Reduces suffering and premature death, lucrative for governments, cost containment/savings, education	Hurts/eliminates jobs, tax on the poor, narrow and unfair, nanny state, promote illicit trade	Old tax modified
Ghana	Singh *et al* 29	SSB, alcohol, tobacco	News media, government documents, NGO reports, legislative proceedings, focus group discussions, key informant interviews	Reduces suffering and premature death, lucrative for governments, product reformation, education	Hurts/eliminates jobs, threat to industry, tax on the poor, promote illicit trade	Old tax modified
Indonesia	Ahsan *et al* 30	SSB, alcohol, tobacco	News media, government documents, focus group discussions, key informant interviews	Reduces suffering and premature death, lucrative for governments, cheap	Hurts/eliminates jobs, threat to industry, meaningless (too small/ineffective), promote illicit trade	New tax created; new tax modified
Nepal	Acharya *et al* 32	Alcohol, tobacco	News media, government documents, legislative proceedings, focus group discussions, structured observation, key informant interviews, desk review	Lucrative for governments, cost containment/savings, pro-poor policy, product reformation, education	Hurts/eliminates jobs, narrow and unfair, meaningless (too small/ineffective), better means to end	New tax created, old tax modified,
Peru	Zuleta *et al* 31	SSB, alcohol, tobacco	News media, government documents, NGO reports, legislative proceedings, industry docs, key informant interviews	Reduces suffering and premature death, lucrative for governments, everyone else is doing it, favourable economic impacts	Hurts/eliminates jobs, threat to industry, tax on the poor, narrow and unfair, promote illicit trade, prices will increase, less competition, tax evasion increase, less investments, no participation from industry	New tax created, old tax modified
Pakistan	Mirza *et al* 33	Tobacco	News media, government documents, NGO reports, focus group discussions, key informant Interviews	Reduces suffering and premature death	Hurts/eliminates jobs, threat to industry, promote illicit trade	Contestation (unresolved)
Vietnam	Dao *et.al*	SSB, alcohol, tobacco	News media, government documents, NGO reports, key informant interviews	Reduces suffering and premature death, lucrative for governments, cheap	Hurts/eliminates jobs, threat to industry, tax on the poor, narrow and unfair, promote illicit trade, tax evasion increase	New tax created, old tax modified

NGO, non-governmental organisation.

## Lessons from individual case studies

Rasheed *et al* analysed political economy factors in health tax design and implementation in Bangladesh. They described barriers to policymaking and implementation process related to tobacco tax. This has led to resistance to increase taxes in the lowest, but largest, segment of the market. The dual role of the Bangladesh government as a regulatory and shareholder of the largest tobacco company weakens the formulation and implementation of taxes.^25^ As one of the highest revenue-generating sectors, tobacco companies wield significant indirect influence within the government and media to shape narratives and weaken the design and implementation of taxes.^26^ However, despite challenges, a strong network and alliance of antitobacco actors including academia, advocacy organisations, non-governmental organisations, the WHO and media are pressuring the government for better tax design and implementation.

Erku *et al* examined framing, moral foundations and health taxes by interpretive analysis of Ethiopia’s tobacco excise tax policy in 2019–2020. They identified the specific framing mechanisms by which a public health coalition stigmatised the tobacco industry to build support for taxation.^27^ This group rejected tobacco industry’s framing of ‘protection of economy and personal freedom’ and cost-benefit assessments by centring and showcasing scientific evidence and lived personal experience of those harmed by tobacco, particularly children. Health advocates framed tobacco taxes calling attention to the financial and psychological costs as well as the productivity losses of tobacco use. Moreover, tax revenue was considered a secondary consideration in this framing.^27^ The formation of a public health coalition and its strategic engagement with policy entrepreneurs accelerated the development of a convincing and affirmative policy narrative leading to the passage of Ethiopia’s tobacco tax.^27^ Despite health advocates deprioritising the revenue benefits of health taxes, the implementation of the tobacco tax was aided by being in the context of a broader national ‘homegrown economic reform’, focused on ‘significant improvements in tax collection’.^28^

In Ghana, Singh *et al* assessed stakeholders’ perceptions on taxing tobacco, alcohol and SSBs.^29^ They reveal how stakeholders’ understanding of health taxes was often limited and unclear.^29^ In contrast to the Ethiopia team, Ghanaian government stakeholders and civil society actors argued in favour of health taxes primarily to generate revenue for health programmes.^29^ Their ability to decrease consumption of harmful products was a secondary aim.^29^ Industry stakeholders opposed health taxes by highlighting their perceived impact on employment, limited revenue generation potential and growth of an illicit market.^29^ The current economic crisis, close relationship between government and industry, lack of accountability (coupled with bribery and corruption) were understood to limit efforts to advance all health taxes.^29^

In Indonesia, Ahsan *et al* conducted a review of media policy debates on the tobacco, alcoholic beverages and SSBs taxes.^30^ They found that while tobacco tax policy has changed significantly, there has been little policy movement on alcohol and SSB taxes.^30^ Leading up to the elections of 2019, media framing intensified, with many political figures adopting clear positions in opposition to taxation.^30^ Policy debates on tobacco and e-cigarette taxation in Indonesia prioritised economic interests over health interests.^30^ This was seen as important, given that Indonesia is one of the tobacco industry’s most important markets. Key opinion leaders’ statements in the media reflected policy contestation surrounding a potential SSB tax, which led to delayed tax implementation.^30^ Support by and leadership from the Ministry of Finance (MoF) was seen as central to the implementation of health tax legislation.^30^

Similarly, Zuleta *et al* examined political and socioeconomic factors shaping the introduction of health taxes on tobacco, alcohol and SSBs in Peru in 2016 and 2018, the years that Peruvian Ministry of Economy and Finance (MoEF) made significant readjustments to tobacco and SSB taxes.^31^ The main arguments for or against health taxes concerned their impacts on the economy, and on health.^31^ This was especially true for government actors, who tended to frame health tax support intrinsically using economic arguments and health-related statements together.^31^ The MoEF, considered a strong, stable and independent institution and empowered with notable autonomy, led to the successful implementation of health taxes despite industry interference, including actions from institutions such as Congress, and political instability.^31^

Acharya *et al* assessed barriers and facilitators to an effective design and implementation of taxes on tobacco and alcohol products in Nepal.^32^ They observed significant regulatory capture with industry exploiting the limitations in the current tax system, including the lack of transparency on how health tax revenues were mobilised.^32^ Moreover, in these debates, the cultural context in which consumption was embedded and the demand for harmful products, despite increasing prices, were also framed as insurmountable barriers to health tax policy change.^32^

Mirza *et al* analysed political institutions and the health taxes regime in Pakistan and identified two key aspects of the country’s political institutions resisting change.^33^ The first barrier to health tax policy were structural issues such as federalism, intraelite conflict, interagency coordination and intra-agency fragmentation in the design and functioning of key institutions.^33^ The other significant barrier to effective health tax policy was the entrenchment of industry interests within government institutions, exploiting these structural issues and taking advantage of gaps in the governance of conflicts of interest.^33^ While tobacco taxes were eventually raised in Pakistan to raise revenue, arguments in favour of health taxes were pre-empted or co-opted prior to being fully formed in deliberative processes.^33^

Dao *et al* conducted a review of policy debates on health taxes, leading up to a proposal to revise the excise tax law in Vietnam in 2023–2024. They found that while health objectives were central arguments to facilitate the excise tax law reform, macroeconomic stability and business protection arguments remained a significant barrier to raising the tax rate to a level needed to reach public health targets. There were also differences in understanding evidence on the harms of taxable products, especially SSBs, in contrast to the case of tobacco.

## Learning across case studies

In these case studies, important themes emerge across frames for and against health taxes. This draws on work that looks at ‘what gets framed’ in the process of creating meaning in policy discourse. The identity and relationship of actors, the content of policy issues and the policy process itself are portrayed in telling ways.^34^ These cases demonstrate how identity is (re)constructed in the policy process. First, Ministries of Finance must often be persuaded that the benefits of taxation outweigh any potential risks. In Ethiopia, the new excise tax was approved only after WHO experts met with the MoF and Ministry of Health to make this case. In Indonesia and Peru, the role and support of these ministries played a crucial role in health taxes adoption and implementation. Second, coalitions are critical to advancing health taxes as they can help convene, combine and unify broad-based arguments in support of health taxes. This is particularly important given the multisectoral nature of NCD prevention and control efforts. For example, a strong public health coalition emerged in Ethiopia and Bangladesh to emphasise the moral dimensions of tobacco taxation in their discussions with other policy entrepreneurs. Third, to build a compelling coalition, stakeholders must be broader than simply health advocates. Health and tax policy experts must engage with broader communities. In Ethiopia, beneficiaries, including children and workers whose livelihoods are affected, briefed parliament helping provide a moral and persuasive account of the potential benefits of health taxes. The presence of prominent people or celebrities, recognisable figures whose life stories serve as relatable public narratives, can also help. Creating a broader coalition beyond health and government actors remains a key strategic challenge for health tax advocates.

The ways in which actors understand their abilities to participate in and shape policy process were important across all case studies; equally important was the way in which research or evidence is produced, sponsored, communicated and deployed. More evidence conducted by what are considered contextually credible sources are needed to help inform debate, as in Indonesia. While the volume of evidence is important, it is not sufficient. The types of arguments research support matters. Media, especially emergent social media, through their dual ability to reflect and shape public opinion, is an important and sometimes underappreciated actor—actors opposing health taxes often have sophisticated media strategies.^35^ All case studies used news media as a source of data, see Indonesia for heavy use and Pakistan for light usage. Media is also essential for coalition-building, advocacy at key strategic times (ie, prior to elections), and by connecting health taxes to other related social policy issues (eg, universal health coverage, child social welfare, education, etc). In this way, health taxes are politically contingent and socially constructed.

The content of health tax proposals as well as the arguments used to support them matters. The international health community consistently reminds advocates that the purpose of health taxes is to improve health and well-being.^36^ Revenue generation is generally considered by many health advocates to be a secondary aim for health taxes.^37^ One of the reasons for this is that more than 80 countries use some form of earmarking for health tax revenue, which is an imperfect instrument of public financial management.^38^ Despite this, empirical evidence on earmarking and how it helps or inhibits health taxes implementation remains limited.^37 38^ Some have argued against earmarking due to concerns about budget rigidity, economic distortion, procyclicality, fragmentation, decreased equity and susceptibility to special interests.^37 38^ Arguments for earmarking are linked to revenue protection, efficiency, accountability, cost awareness, flexibility and public support.^37 38^

In several instances (in Ethiopia, Ghana, Indonesia, Nepal and Peru), highlighting the revenue aims of health taxes was a major contributor to the passage and advancement of health tax legislation. This corresponds to research on SSBs in the USA suggesting public opinion moves to a favourable position only when the revenue generated by a potential tax is clearly directed towards some specific (social) aim, such as education or healthcare.^39–41^ Similarly, others have observed that mistrust in governments’ use of revenues, or lack of transparency in the implementation or revenue allocation process, are stubborn barriers to health tax policy.^12 13^ Findings from this context-specific cross-country research in LMICs suggest that there is no clear trade-off between health and economic framing of health tax policy proposals, nor is there a straightforward way to handle concerns around earmarking. More research is needed to contrast efforts between high-income countries (HICs) and LMICs and clarify the contexts in which these two aspects of health taxes resonate most fully.

This research can help illuminate the likely types of arguments to support or oppose health taxes. The most common arguments in support of health taxes were that they reduce suffering and premature death (n=7/8 studies) and are lucrative for governments (n=7/8) (table 1). Secondary arguments in support of health taxes found in half (4/8) of these studies were that they represent an opportunity to contain the rising costs of NCD medical care and the associated savings to the health system, they encourage product reformulation, and they could be directed towards other underfunded sectors such as education and social health insurance (table 1). Arguments that did not surface as readily were arguments based on benefits to the economy or based on international precedent.

Arguments against health taxes largely focused on their potential to hurt the economy or eliminate jobs (n=8/8 studies) and promote illicit trade (n=7/8 studies) (table 1). This was perhaps due to the focus of tobacco taxes in all eight countries (compared with SSBs and alcohol in four), where illicit trade has been a persistent source of concern, including its potential to lead to increasing levels of crime. Less frequently, arguments focused on threats to associated industries and highlighted the position that health taxes are narrow and unfairly target a specific segment of industry. Arguments that health taxes hurt the poor featured more prominently than arguments that health taxes represent pro-poor policy.

Health tax frames in these eight studies largely reflect national-level power struggles within government and between government and private industry. Furthermore, in none of our countries did a single frame dominate. Framing is contested, with different kinds of arguments linked to different moral positions. We propose that it is not the strength of a given frame, but the combination of different frames that allow ideas to potentially resonate with a broader coalition of interests, enhancing actors’ positions in favour of or against health tax policy. This has the potential to attract or compel political actors to engage and can help shape, or in fact create, political windows of opportunity to advance health tax policies. More research could be done to explore less common frames as well as consider ongoing policy transfer on health taxes among countries as well as the interaction between policy frames and the broader political economy and context for policy-making

In case of study countries, advocates surprisingly made little use of arguments in favour of the economic benefits of taxation in terms of providing resources for government budgets and/or social investments. One could conclude that making an investment case for the distribution of health and economic benefits from health taxes could be a priority. Modelling studies and evaluations from other countries can help supply these arguments.^42^ Similarly, learning from and citing the experience of regional peers can help establish precedent and credibility for nascent health tax proposals. Efforts to benchmark health taxes could be strengthened and expanded to accelerate progress; for example, Tobacconomics produces a cigarette tax scorecard.^43^ Existing transnational organisational learning can be further supported to develop a deeper and wider community of experts within an international policy network to advance health tax design and implementation.^44 45^ While recognising the continued limitations of tobacco tax implementation,^46^ learning from global tobacco control policy will help confront the commercial determinants of health.^47 48^

Much debate focuses on the economic threat to local industry. Yet, it remains unclear why few arguments surfaced that characterise multinational corporations as external threats to the sovereign rights and health of citizens. Civil society organisations with a regional or international remit may be well-placed to expand existing and initiate new advocacy campaigns. Finally, it seems health advocates can enhance their claims about the health benefits of taxes by drawing disproportionately on and centring the health of vulnerable members of society such as children and the poor. Similarly, building on the tobacco experience in Ethiopia, graphic appeals to bodily harm associated with NCDs could be used to great effect. In this way, an arsenal of different kinds of frames can help position health tax reforms as part of an inevitable global movement transcending domestic policy disputes and underpinned by an increasingly sophisticated evidence base.

## Lessons for global efforts and the importance of social mobilisation

Framing strategies have the potential to locate arguments within wider ideological movements, thus enhancing their credibility, changing the nature of the issues and potentially resolving protracted policy controversies. Indeed, the power this confers to individuals, organisations and institutions is what makes framing a competitive pursuit in politics.^49^ Drawing on the social movement literature, frames resonate when they align with circulating value structures.^50^ For example, as in the case of the Philippines, health taxes can be framed as equity measures that improve health and wellness and contribute to domestic resource mobilisation to reduce and fund the costs of care accelerating efforts to achieve universal health coverage.^51^ Literature on framing in policy conflicts has demonstrated how reconnecting aspects of a frame to comparable beliefs can help broaden their acceptability.^52^ In this way, health taxes can be framed as not only measures to improve health but broader contributions to planetary health and sustainable development, including multisectoral benefits to the natural environment, consistent with the Sustainable Development Goals.^4^ Finally, by drawing on the framing literature in communication, a plurality of frames can provide insight into which kinds of arguments resonate with the public as well as with policy elites.^53^ This is a key source of power in political settings. Moreover, which arguments resonate may also depend on larger structural forces such as economic recession, international migration or global integration, many of which can change rapidly. In this way, health taxes can be positioned symbolically as social commitments to building more resilient health systems that can better absorb future crises such as pandemic disease, war and natural disasters. In so doing, researchers, advocates and policymakers alike can work together to learn from the collective experience of taxation and the appropriate distribution of its benefits for generations to come.

## Agenda for action

Defining roles and responsibilities of different stakeholders and collaborative advocacy play a crucial role to address industry interference for health taxes. For example, finance and tax experts are obvious and necessary allies and need to be part of health taxes advocacy coalitions. Coalitions capable of mobilising across the broader commercial determinants of health can be built at the local, national and international levels. Multilateral development actors can play a significant role in promoting well-designed health taxes. For example, through efforts such as the Interagency Working Group on Health Taxes, UN agencies (eg, WHO) and international organisations (eg, World Bank, International Monetary Fund, etc) have been developing a series of evidence-informed policy packages related to health taxes that help policymakers consider their implications for employment, revenue generation, agriculture, illicit trade and social inequality. These information dissemination and strategic support initiatives need to be accompanied with sufficient funding for contextually-nuanced/politically-aware technical assistance, monitoring and implementation support for sustainability.

Policy alignment is key to deliver effective action plans for health taxes. Because countries are dynamic, this is uniquely tied to political circumstance. Developing comprehensive national frameworks can achieve greater policy coherence, partnerships and stronger surveillance systems. It is important to note that government itself may be divided, with some ministries/departments aligned with industry. Building support for health taxes; therefore, is a continuous process of building internal consensus in ways that reflect citizen’s goals. Framing strategies may differ between government agencies, between government and industry, or between governments of neighbouring countries. Governments can also improve their capacity to counter the framing strategies used by industry by setting rules about their ability to interfere with the political process, undertaking due diligence and having transparent processes in decision-making. Article 5.3 of the WHO Framework Convention on Tobacco Control provides an example for other industries, especially if it was to include a binding code of conduct.^54^ To address the extensive lobbying power of industry, international institutions as well as public health professionals and the scientific community can provide critical support to governments by engaging in improving information dissemination for advocacy and policy development. Civil society groups and non-government organisations can be advocates for proactively facilitating the health literacy of parliamentarians.

By working with public health professionals and health scientists, a public health coalition can exert pressure on governments to act in the public interest. Awareness, attention and progress in addressing harm are driven by interactions between issue characteristics, policy environment and global health networks.^21^ Therefore, creating and maintaining widespread consensus about effective policies for health taxes by combining local evidence with effective advocacy at global levels would be able to support countries to build strong institutions and leadership on health taxes.

## Agenda for research

The case studies examined here reveal a number of gaps that merit further research. First, there is insufficient attention to how the political economy of health taxes is complicated by the transnational character of dominant industrial corporations. Political economy analysis to understand how industrial corporate framings at the global, regional and country level influence health tax policy is needed. Moreover, empirical research exploring the context-specific power and institutional dynamics as well as the political complexity of designing, framing and implementing health taxes is also crucial for better understanding the processes and policy dynamic of health taxes, especially in LMIC settings. The political economic analysis of health taxes needs to be linked with commercial determinants of health to have a broader understanding of economic, social and public health benefits to overcome powerful commercial interests.^3 23^

Second, none of the case studies deeply considered the mechanisms for tax collection in their respective country and their maturity as a factor in the policy debate for health taxes or substantively considered the relative merits of hypothecation. There is a need for greater engagement of finance ministries and tax experts themselves in the development and execution of political economy analysis of health taxes, with greater attention to the science and methodologies of taxation.

## Conclusion

This research can help accelerate new understandings of health taxes as a mechanism to protect the health and human rights of people around the world and mobilise domestic revenue for social programmes. More research considering the political economy, framing and how health taxes are linked to the broader commercial determinants of health should be conducted in LMICs to add to the early evidence presented here, in addition to greater engagement with the finance sector and taxation experts. More research is particularly needed on framing, especially on particularly on how and which frames work best where, when and why. While we found no single frame which dominated in the eight LMICs where we conducted research, a plurality of frames was understood to shape the implementation (or lack thereof) of health tax policy. Arguments for and against health taxes were similar across countries but differed in their priority and emphasis. While earmarking remains a contested approach to health taxes, similar to HICs, there is some evidence that populations in LMICs (and thus policymakers) are deeply concerned about how and where tax revenues are spent and invested. While health taxes are no panacea, if designed appropriately, they can help make the world a fairer place by helping consumers make better decisions and governments serve as effective stewards of population health. To accelerate progress, health tax advocates will need to deploy multiple frames (including perhaps frames not yet widely used) and develop broader coalitions that extend beyond the nation state and the health sector.

## Data Availability

Data are available upon request.
